# Weak connections form an infinite number of patterns in the brain

**DOI:** 10.1038/srep46472

**Published:** 2017-04-21

**Authors:** Hai-Peng Ren, Chao Bai, Murilo S. Baptista, Celso Grebogi

**Affiliations:** 1 Shaanxi Key Laboratory of Complex System Control and Intelligent Information Processing, Xi’an University of Technology, Xi’an 710048, China; 2 Institute for Complex System and Mathematical Biology, University of Aberdeen AB24 3UE, United Kingdom

## Abstract

Recently, much attention has been paid to interpreting the mechanisms for memory formation in terms of brain connectivity and dynamics. Within the plethora of collective states a complex network can exhibit, we show that the phenomenon of Collective Almost Synchronisation (CAS), which describes a state with an infinite number of patterns emerging in complex networks for weak coupling strengths, deserves special attention. We show that a simulated neuron network with neurons weakly connected does produce CAS patterns, and additionally produces an output that optimally model experimental electroencephalograph (EEG) signals. This work provides strong evidence that the brain operates locally in a CAS regime, allowing it to have an unlimited number of dynamical patterns, a state that could explain the enormous memory capacity of the brain, and that would give support to the idea that local clusters of neurons are sufficiently decorrelated to independently process information locally.

Much progress has been made in recent decades for the understanding of brain function^1^,^2^. Brain function is determined by the way in which neurons are connected (both the topology of the neuron network and the synaptic strength among the neurons), and the intrinsic dynamics of the neurons forming the brain. Typically, one obtains an approximate course-grained information of the brain by inference from time-series measurements. The EEG signal^3^, used to obtain information about a local state of the brain, has been widely utilized in medical diagnosis and analysis due to its many advantages: non-invasive, low cost implementation, high-temporal analysis, tolerant to subject movements and so on. For that reason, the characterisation of this signal and the understanding of which kind of neuron dynamical behaviour produces an EEG signal have become an area of intense research^4^. One of the greatest challenges in brain research, which relies also on EEG measurements, is the understanding of memory formation. The binding hypothesis^5^, referring to a process in which perception happens by making different cognitive and memory areas of the brain synchronous (bind), is a representative class of work in brain research that also relies on EEG measurements. EEG data, as well as data obtained from several other methods, is thus used to shed light into the binding connecting network structures^6^,^7^,^8^,^9^,^10^ and the strengths of the neuron synapses in the brain. Modelling EEG signals is essential to understand the anatomy and histophysiology of the brain, and therefore provide support to medical imaging analysis and to the development of neuroscience^11^. However, consensus about what is the topology of the brain or its synaptic *modus operandi* is still an open problem. On the one hand, the understanding of signals from living brain is limited by non-invasive biological techniques. On the other hand, the available methods to infer the brain connectivity structure from measurements, such as EEG^12^, can only provide a rough estimate of the large-scale structure of the brain, little being known about the connectivity of the local clusters of neurons generating EEG signal.

To elucidate the fundamental dynamical mechanisms behind observable brain behaviour, there has been a lot of research based on simulations of neuron networks and their behaviour as a function of the intensity of the coupling strengths^13^,^14^,^15^,^16^,^17^,^18^,^19^,^20^ and the connecting topology^21^,^22^,^23^,^24^. Under strong coupling strengths, neuron networks of equal neurons support the appearance of complete synchronisation (CS)^17^,^18^,^19^,^20^, as shown in Fig. 1. In this case, all the neurons in the network tend to have the same oscillatory dynamic behaviour. Thus, these neurons could be equivalent to a single neuron, and thus being not amenable to exhibit complex oscillatory brain patterns. With the decrease of the coupling strength, neuron networks can exhibit some well known weak forms of synchronisation^14^, such as phase synchronisation (PS)^16^, partial phase synchronisation (PPS)^14^, bursting phase synchronisation (BPS)^14^, and collective almost synchronisation (CAS), as shown in the intervals (2) and (1) of the Fig. 1, respectively. The CAS, a phenomenon where nodes are weakly connected, is characterised by the existence of local clusters of neurons possessing roughly constant local mean fields, consequence of neurons being very weakly connected. It terminates when local mean field starts to oscillate. The conventional wisdom is that the neurons are completely disordered when coupling strength is very weak. However, a recent research work^25^ shows that CAS is present in complex networks for very weak coupling strengths. In this situation, the neuron networks can process an infinite number of possible oscillatory patterns. CAS phenomenon is thus a plausible explanation for the existence of local cluster of neurons that are sufficiently decorrelated to independently process information locally. Those infinitely many patterns that can appear in simulated neural networks working under the CAS regime could provide the basis and shed light into the process of memory formation in the brain.

Whereas the small-scale and global connecting topologies are still a major unknown, much is understood about the intrinsic dynamical features of neurons, i.e., their equations of motion. The pioneering work on neuron models was done by Hodgkin and Huxley^26^. They proposed a fourth-order model to study dynamical behaviour of individual neurons. To simplify the model, second-order models, such as the ones by FitzHugh-Nagumo^27^ and Morris-Lecar^28^, were proposed. However, the second-order models are not able to reproduce some phenomena, such the triggering of a set of stable firings^29^. Hence, a third-order model, referred to as Hindmarsh-Rose (HR) model, was introduced to solve that problem^30^,^31^,^32^,^33^,^34^. For our analysis, we consider the HR model.

In this work, we are aimed at explaining the dynamical and structural fundaments of the neuronal networks responsible for generating experimental EEG signals. Our goal is to shed light into the characteristics that a simulated neuron network (i.e., coupling strengths and topological structure) needs to have in order to model experimental EEG signals. We show that networks operating in a CAS regime optimally model EEG signals, manifesting the activity of a local part of the outer layer of the brain. Our results suggest that locally the brain has neurons that are only weakly connected. Moreover, since the CAS regime is weakly dependent on the neuron connectivity, our results imply that at smaller scales the synapses strength is the most relevant parameter for brain behaviour. Because the CAS regime allows a network to exhibit an infinite number of patters, our work also provides an explanation about the brain mechanism for its enormous memory capacity. Finally, our model of the EEG signal is based on similar protocol to produce weighted average outputs of complex networks that integrate information of various clusters to produce a logical “intelligent” output. Consequently, our work also suggests that information in the brain is being locally processed by independent local clusters of neurons.

In the following, we provide the organisational presentation of our main results in this paper:

(1) We show in Sec.2 that the CAS phenomenon occurs in neuron networks with both chemical and electrical synapses.

(2) We show that a mean field from a random cluster of neurons in the simulated networks exhibits CAS, optimally reproducing experimental EEG signals (in Sec.3). This result supports the argument that CAS is present in localised regions of the brain, suggesting that local clusters of neurons does not have strong synaptic strengths. Knowing that CAS allows for the formation of an infinite number of collective clusters and patterns, the presence of CAS in local regions of the brain could be advantageously used as a memory reservoir.

(3) Neuron networks with both linear (electric) and nonlinear (chemical) couplings, as known to exist in our brain^32^,^33^,^34^, reproduce surprisingly well the experimental EEG signals for the coupling strengths in the range responsible to produce the CAS regime.

## Criteria to exhibit the CAS phenomenon in neuron networks with both electrical and chemical synapses

### Neuron network

The Hindmarsh-Rose(HR) neuron model^35^ is the following:


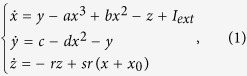


where *x* represents the membrane potential, *y* is the recovery variable associated with the fast current of *Na*^+^ or *K*^+^ ions, *z* is the adaptation current associated with the slow current of, for instance, *Ca*^+^ ions, *I*_*ext*_ is the externally applied current that mimics the membrane input current for biological neurons, *r* is a small parameter that governs the bursting behaviour, and *x*_0_ is the resting potential. The following choice of system parameters, *a* = 1, *b* = 3, *c* = 1, *d* = 5, *s* = 4, *r* = 0.005, *x*_0_ = 1.618 and *I*_*ext*_ = 3.25, yields the HR neuron model to exhibit a multi-time-scale chaotic behaviour characterised by spiking bursting^36^.

**Figure 2:**
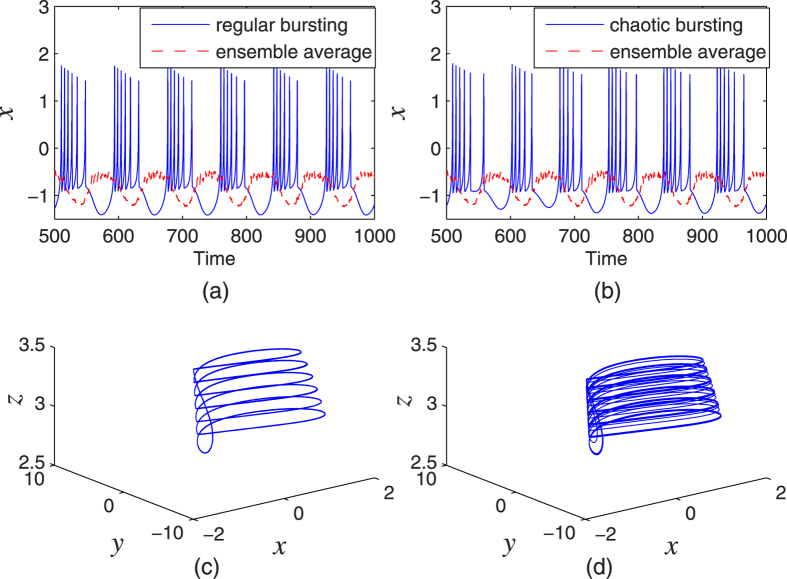
Various dynamical behaviours of the HR neuron in the neuron network, when the CAS phenomenon is present. Panels (a,b) show the membrane potentials of regular bursting and chaotic bursting, respectively. The red dashed lines in panels (a,b) are the ensemble average of the network. Panels (c,d) show the phase space plot of regular bursting and chaotic bursting, respectively.

The dynamical equations of an HR neuron network with both electrical and chemical synapses is the following:


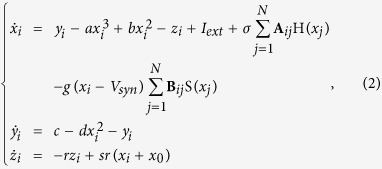


where 
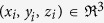
 are state variables of the neuron *i* with random initial conditions, *i* = 1, 2, …, *N. N* is the number of neurons in the network. 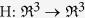 is the internal coupling function among the neurons in the network; we consider H(*x*_*j*_) = *x*_*j*_ − *x*_*i*_. *σ* and *g* are electrical (linear coupling strength) and chemical synapses (nonlinear coupling strength), respectively. The matrix **A**_*ij*_ represents the Laplacian matrix for the electrical synapses; the adjacency matrix **B**_*ij*_ describes the topology of the chemical connections, thus 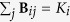, where *K*_*i*_ represents the number of chemical connections that neuron *i* (the post-synaptic neuron) receives from all the other *j* neurons (pre-synaptic neurons) in the network. The chemical synapse function is modelled by the sigmoidal function


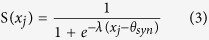


with *θ*_*syn*_ = −0.25, *λ* = 10, and *V*_*syn*_ = 2.0 for excitatory and *V*_*syn*_ = −2.0 for inhibitory neurons^36^. In Fig. 2 we show examples of dynamical behaviours of the membrane potentials *x* (solid line) and ensemble average of the neuron network (dashed line) when the CAS phenomenon is present, where *I*_*ext*_ = 3.25, *N* = 1000, *σ* = 0.001, *g* = 0, and other parameters are the same as the ones used to simulate the HR neuron in Eq. (1). Simulations were performed using Matlab Simulink.

### Revisiting CAS phenomenon in networks whose neurons are coupled with only electrical synapses

The CAS phenomenon was first described and analysed in ref. 25. It describes a universal way of how patterns could appear in complex networks for weak coupling strengths. The local mean field of node *i* is defined as:


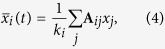


*k*_*i*_ is the electrical coupling degree of node *i*. The expected value of the local mean field is defined as


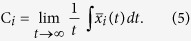


The CAS patterns of node *i* is described by:


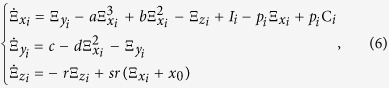


where 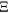 are the three-dimensional CAS patterns, *p*_*i*_ = *σk*_*i*_ is the product of the coupling strength *σ* and the degree of node *i*, i.e., *k*_*i*_, for linear coupling. Equation (6) is derived from a local mean field approximation describing approximately the evolution of a cluster of neurons that have all the same connectivity degree.

**Figure 3:**
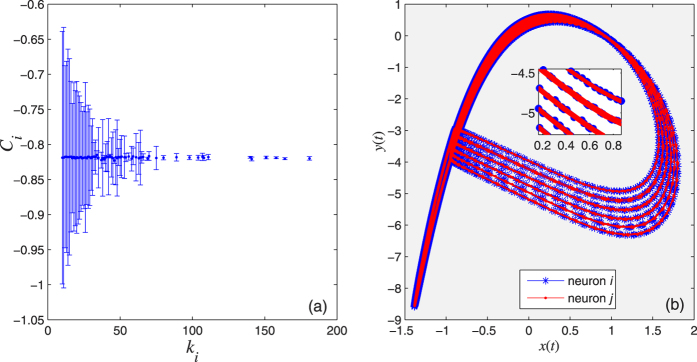
The two criteria for the existence of the CAS phenomenon; we choose a scale-free network (a network whose degree distribution follows a power law) with *N* = 1000, *σ* = 0.001, *g* = 0, *I*_*ext*_ = 3.25 and *r* = 0.005. (**a**) Expected value of the local mean field of node *i* against its degree *k*_*i*_. (**b**) The CAS patterns for the neuron *i* and another neuron *j* with degree *k*_*i*_ = *k*_*j*_ = 25. The inset is a blow up.

There are two criteria for the network to present the CAS phenomenon. They are as follows:

- **Criterion 1:** The Central Limit Theorem should be applied, i.e., 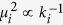. Therefore, the larger the degree of a node, the smaller the variance of the local mean field 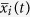 about its expected value C_*i*_, as shown in Fig. 3(a). Here, 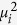 denotes the variance of 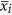.
- **Criterion 2:** There must be a CAS pattern described by a stable periodic orbit, to which two locked neurons can demonstrate phase or delay synchronisation (or other functional synchronisation^25^), as shown in Fig. 3(b).

**Figure 4:**
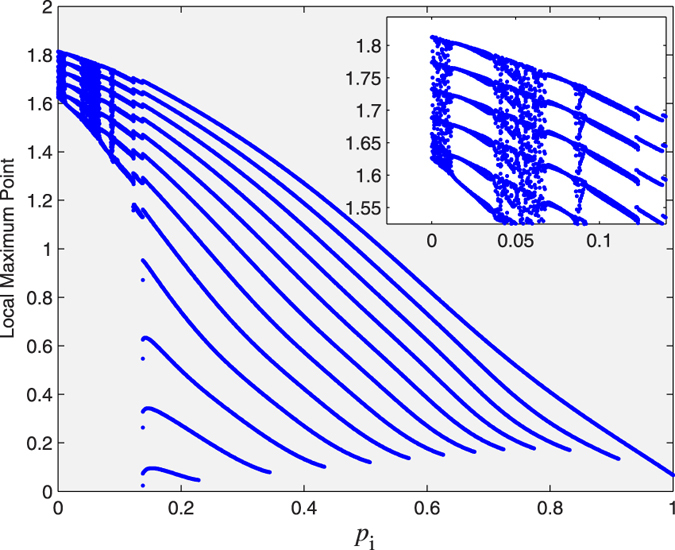
A bifurcation diagram of CAS patterns with the variation of *p*_*i*_ in the neuron network by only considering electric synapses.

The different CAS patterns are obtained by varing *p*_*i*_, which can be analysed by using a bifurcation diagram as shown in Fig. 4, where we plot the local maximal points of the CAS patterns. We find that when 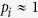, there is only one pattern in the neuron network. With the decrease of *p*_*i*_, the patterns have a period-adding transition from period one to period two, period three, and so on, followed by an infinite period doubling cascade leading to a chaotic state, the latter occurring when *p*_*i*_ is close to 0, as shown in the inset plot. Therefore, this is the bifurcation scenario toward an infinite number of patterns.

Notice that the existence of the CAS pattern only requires an approximately constant local mean field *C*_*i*_, and it does not depend on the topology of the network. So, our subsequent analysis is reproducible in any arbitrary network, as long as there is a sufficient number of well connected nodes. Our numerical results suggest that a minimum degree of about 10 is necessary to create a constant *C*_*i*_. Equation (6) is an approximate equation to describe the evolution of nodes of the network with weak coupling. If two nodes in the network would be in the CAS regime, they would have their dynamics described approximately by Eq. (6). If this equation would describe a stable periodic orbit, as in the requirement for the CAS regime, the real trajectory of these nodes would be a perturbation around the same stable periodic orbit. The trajectory would never escape the periodic orbit, which would make these nodes to become phase synchronous, after a time-delay would be taken into consideration.

### The CAS phenomenon in networks whose neurons are coupled with both electrical and chemical synapses

Due to H(*x*_*j*_) = *x*_*j*_ − *x*_*i*_, the electrical coupling term of the first state variable can be rewritten as


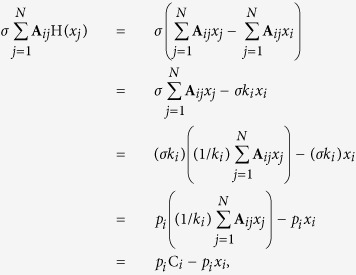


where *p*_*i*_ = *σk*_*i*_, 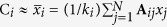.

The chemical coupling term 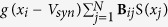 can also be rewritten as


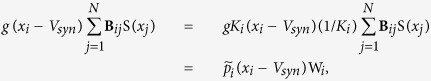


where 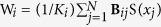, 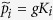, *K*_*i*_ is the chemical coupling degree of node *i*. Hence, the CAS patterns with both coupling are given by


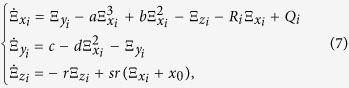


where 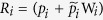, 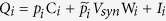. To illustrate the presence of the CAS phenomenon, we consider a scale-free network, formed by *N* = 1000 neurons, with both electrical and chemical couplings. The simulations show that the two criteria for the CAS phenomenon are also satisfied in this neuron network with both electrical and chemical synapses.

**Figure 5:**
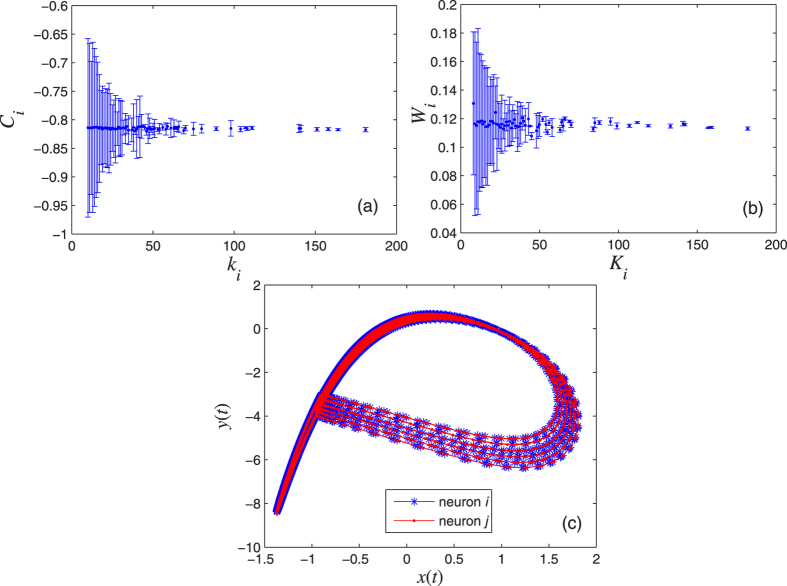
Results validating the two criteria for the neurons to exhibit the CAS phenomenon; we choose a scale-free network with *N* = 1000, *σ* = 0.001, and *g* = 0.001. (**a**) Expected value of the local mean field of node *i* with respect to its electric coupling degree *k*_*i*_. (**b**) Expected value of the local mean field of node *i* with respect to its chemical coupling degree *K*_*i*_. (**c**) The CAS pattern for neurons *i* and *j*, both with electrical coupling degree *k*_*i*_ = 20 and chemical coupling degree *K*_*j*_ = 22.

- Figure 5(a,b) show that the Central Limit Theorem can be applied, which happens when the variables are weakly correlated. The error bars indicate the variance of C_*i*_ and W_*i*_, behaving as ∝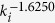 and ∝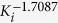, respectively.
- In Fig. 5(c), the CAS pattern describes a stable periodic orbit. The node trajectory can be considered a perturbed version of its CAS pattern.

**Figure 6:**
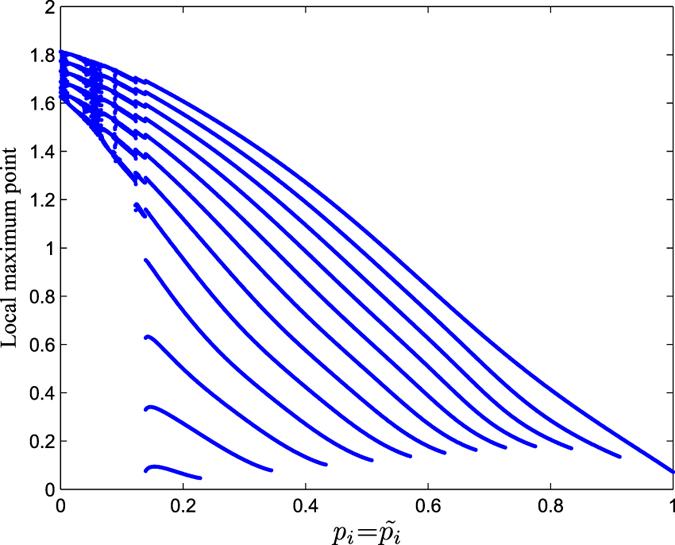
The maximal values of the periodic orbits of Eq. (2) are shown in the bifurcation diagram with both chemical and electrical couplings, where **A**_*ij*_ = *k*_*i*_*I* − **B**_*ij*_, *σ* = *g* = 0.001.

A bifurcation diagram is shown in Fig. 6, where we set **A**_*ij*_ = *k*_*i*_*I* − **B**_*ij*_ for simplicity, where *I* is the diagonal identify matrix. For the case where 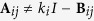, separate bifurcation diagrams for electrical and chemical couplings can be obtained. From Figs 5 and 6, a conclusion is drawn that the CAS phenomenon is typical in neuron networks with both weak electrical and chemical couplings.

## Methods

### Modelling the EEG signal by simulated neural networks

According to ref. 25, if we wish the CAS phenomenon to be seen in a physiology experiment^37^, we need to set up the experiment to probe the firing behaviour of pairwise neurons with the same degree, or equal weighted degree if neurons are connected with non-uniform coupling strengths. As a signature of CAS, these neurons exhibit phase or delay synchronous behaviour. It is difficult to measure individual neurons in living tissue, not to mention searching for neurons with equal degree, or with similar weighted degree connections. Therefore, in this work, we propose to study another signature of CAS, an output function defined by weighted mean field measurements of a cluster of randomly chosen neurons in the network. Our goal is to show that this output function does share remarkable similarities with typical output local mean field measurements of the brain, i.e., the EEG.

EEG is a graphical record of electrical activity of a localised region of the brain, obtained by placing an electrode in a specified location of the head, such as the scalp and subdural placements. The electrode collects a signal that is a function of a weighted sum of postsynaptic potential of nearby neurons in a localised region in the outer layer of the brain. This signal is then compared to a reference electric potential in another region of the brain, and the potential difference is a bipolar signal called EEG. Therefore, EEG is a bipolar electric signal related to a measure of the potential of a local cluster of neurons as compared to the potential generated by another region. Suppose that there are *n* neurons, one fraction influencing the measurement at an electrode and the other fraction influencing the potential produced at the probe measuring the reference signal. Each of these neurons produce a potential 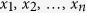. We then model the EEG experimental signal, represented by 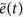, by the following linear sum,


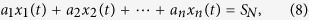


where 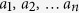 are the linear weights that allow a mapping between the sum of the neuron potential and the EEG signal. We want to find the set of linear weights that minimises the difference 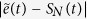. Linear models based on a weighted sum of membrane potentials from simulated neuron network have been previously proposed to model EEG signals^4^. Our novel contribution is to first determine under which network conditions the models optimally fits the EEG signal, and to withdraw general conclusions about the characteristics of neuron network, capable of reproducing these signals, i.e., the parameter conditions of the neurons and their connectivity. Assuming that the mapping of Eq. (8) describes well the link between electric potential and EEG signals, our goal is to show that an experimental EEG signal can be well reproduced by a weighted sum, i.e., *S*_*N*_, but where *x*_*i*_ in Eq. (8) is the membrane potential generated by the simulated neuron network in Eq. (2). In this interpretation, the simulated neural network is actually representing two local areas of the brain, one close to where the EEG main probe is placed and another where the reference probe is placed.

Equation (8) can be written in a matrix form for all *t* ≥ 0, as


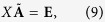


where 
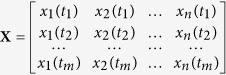
 is a matrix comprised of the membrane potential of the simulated neurons at sampled time *t*_*i*_, 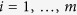. 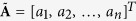 is the weight vector, 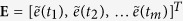 is the sampled EEG signal, and *m* is the EEG signal data length. By using pseudo-inverse method^38^, we can derive 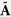 and define the fit deviation as


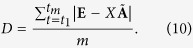


The solution of Eq. (9) provides us with the constant parameters of our EEG model in Eq. (8). We choose randomly a group of nodes (*n* neurons) in the HR network in order to model the EEG data, and calculate the deviation *D*. Observability theory^39^ shows that in nonlinear networks a produced sample of nodes in a network carries information about the whole network. Notice that the approach proposed, which searches for a solution of Eq. (9) by the pseudo-inverse method, is a typical method used to compute a ‘best fit’ (least squares) solution and can be seen as a practical use of the so called computing reservoir paradigm^40^. This paradigm is an approach to calculating the weights of an output weighted average in order to reproduce EEG. The calculation of the weights employs other techniques besides the pseudo-inverse, which minimises the best mean square fitting between the target signal (in this case, the EEG signal, 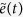), and the weighted average (represented by *S*_*N*_(*t*)). The success of our model to reproduce the EEG signal thus suggests that the brain processes information locally by a small-scale cluster of neurons.

Notice that our model in Eq. (8) is a bottom-up model. We depart from microscopic dynamical properties that lead a network to have CAS regime in its microscopic components to model the EEG experimental signal, a higher level signal, generated by a complicated composition of signals coming from several places in the subject head. As such, our modelling comparison must be done at the level of the signal. In other words, validation of the accuracy of our modelling Eq. (8) by Eq. (10) is at the core of the analysis to be presented in the following.

### The EEG signal approximation using the network with only linear coupling

**Figure 7:**
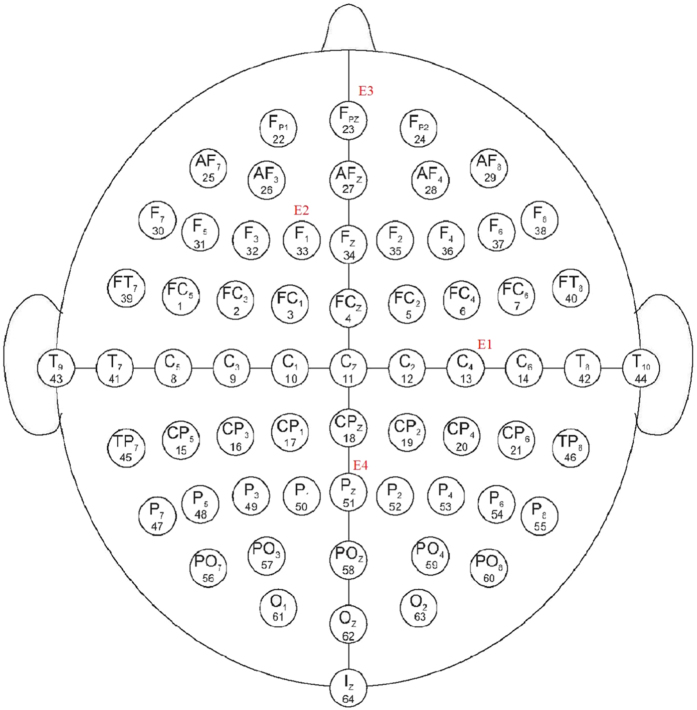
The four electrode locations for C4, F1, Fpz and Pz, the reference probe is placed in earlobes.

**Figure 8:**
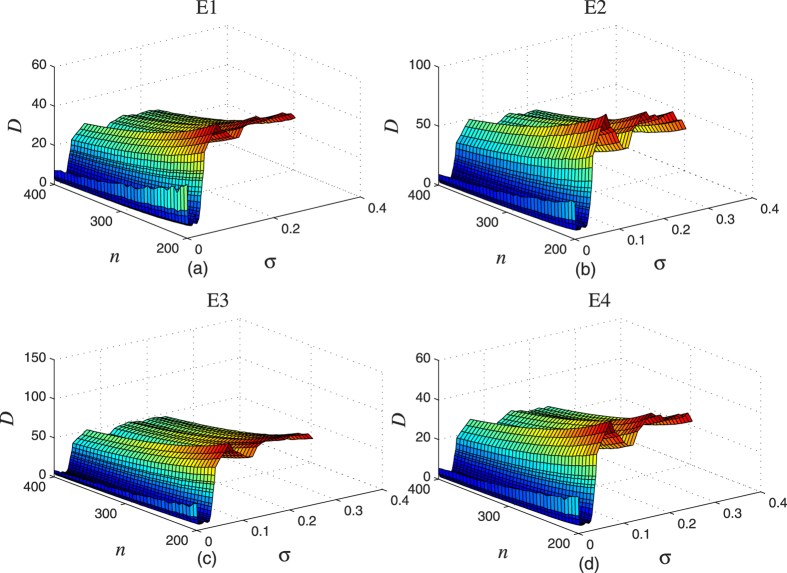
The fitting results for our model of the EEG signals at different electrode positions, where *σ* is the linear coupling strength of the neuron network, *n* is the number of neurons randomly selected for the modelling. The local mean field in the *S*_*N*_ of Eq. (8) is calculated considering *n* = [200, 400] randomly selected neurons in the scale-free network with *N* = 1000 Hidmarsh-Rose neurons; *D* shows the minimum value obtained in 200 tests. Subplots (**a**–**d**) are the result for E1, E2, E3, and E4, respectively.

We consider an HR network to model the actual EEG data at four brain locations. The four experimental EEG data from clinical trials were measured continuously in 64 channels at a sampling rate of 100 Hz and lasting 10 s, i.e., the EEG signal includes 1000 sampling points with the sampling interval equal to 10 ms. The electrode locations are C4, F1, Fpz and Pz, corresponding to the EEG data sets defined as E1, E2, E3, and E4, respectively, as shown in Fig. 7. E1 and E2 were measured in epilepticus state, and E3 and E4 were measured in sleep state. The neuron signals are obtained by using Eq. (2) with a scale-free network; it consists of *N* = 1000 Hidmarsh-Rose neurons with uniform linear (electrical) coupling *σ* = 0.001 and *g* = 0. The time sequences of the selected neurons are sampled using the interval equal to 10 ms, as well. The parameters of the neuron model are the same as in Eqs (1) and (2). Then Eq. (9) is used to fit by Pseudo-Inverse method. Repeated tests are performed, the goodness of fitting results are shown in Fig. 8, where *n* represents the number of neurons randomly selected for data modelling in Eq. (9), *D* is the smallest deviation in 200 tests, *σ* is the linear coupling strength in Eq. (2).

In the Fig. 8(a,b), the results of the modelling for E1 and E2 are given, and the results for E3 and E4 are given in Fig. 8(c,d), respectively. Notably, we can gather some interesting observations from the results in Fig. 8. Firstly, when the coupling strength is equal to zero, the deviation is relatively large. Therefore, if we tried to model the EEG data using time-series generated by disconnected (and therefore, decorrelated) neurons, we would not have achieved a so good fitting as the one when it is done by considering time-series trajectories obtained from a neuron network of weakly connected nodes, possessing the CAS phenomenon. The interpretation of this result is that the EEG signals are not possible to be generated out of a mean field of a set of deterministic variables that are decorrelated. Therefore, the variables generating the local weighted mean field producing the EEG signals must be weakly correlated. Secondly, for fixed *n*, when the coupling strength increases to the range corresponding to CAS, the deviation is the smallest. These two observations suggest not only that local clusters of the brain could be functioning in a CAS state to generate the EEG signals, but also that the computing reservoir paradigm, applied to reproduce an EEG signal, reaches its best performance if complex networks operate in a CAS regime. Therefore, the level of correlation between the deterministic variables must be weak. Thirdly, when the coupling strength increases further to the range leading to the onset of weak and strong forms of synchronisation, the deviation increases accordingly. In summary, the EEG cannot be reproduced by local weighted mean field of variables strongly correlated. When the CS occurs, the deviation is the largest. Finally, we notice that, with the increasing in the number of neurons, the deviation decreases, but it is not as significant as the change for the coupling strength. The minimum degree of scale-free networks grows with the square root of the size of the network, and an increase in the coupling *σ* increases the stability of the CAS pattern (making it more likely to occur). This provides another evidence that the reason for the local mean field (*S*_*N*_) to model the EEG signal (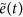) is the presence of the CAS phenomenon in the network, which is strongly dependent on *σ* and moderately dependent on the size of the network. Notice that since the minimum degree grows with the square root of the number of nodes, the range of *σ* values that induces CAS does only moderately change in the interval considered, 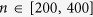. Notice also that the larger the number of neurons considered in the simulations, the smaller the coupling strength needed for CAS to emerge. For the EEG signals from other positions, we obtain similar results.

**Figure 9:**
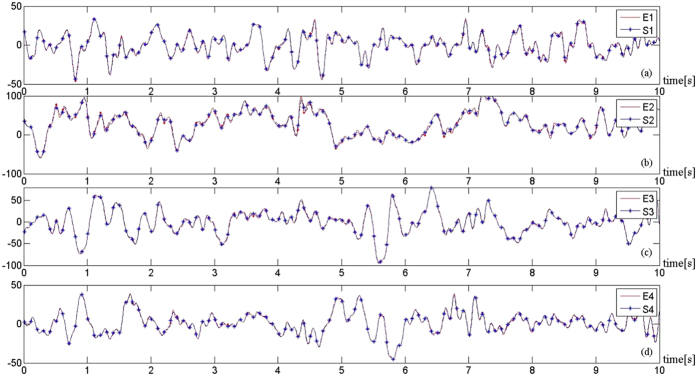
The goodness results at different electrode positions using coupling strength *σ* = 0.001 and the number of neurons *n* = 400; randomly selected to calculate the modelled EEG signal *S*_*N*_. *S*1, *S*2, *S*3, and *S*4 are the best fitting (minimum *D*) within 200 tests for *E*1, *E*2, *E*3, and *E*4, respectively.

To show how good *S*_*N*_(*t*) approaches 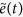, the fitting for the model in Eq. (8), are shown in Fig. 9(a–d), corresponding to the results for E1, E2, E3, and E4, respectively. The actual EEG is shown in red full lines and the model signal obtained by the fitting from Eq. (9) by star marked blue lines, and denoted by S1, S2, S3, and S4, respectively. The number of neurons used in our simulations to produce the results in Fig. 9 is 400, the linear coupling strength is 0.001.

**Figure 10:**
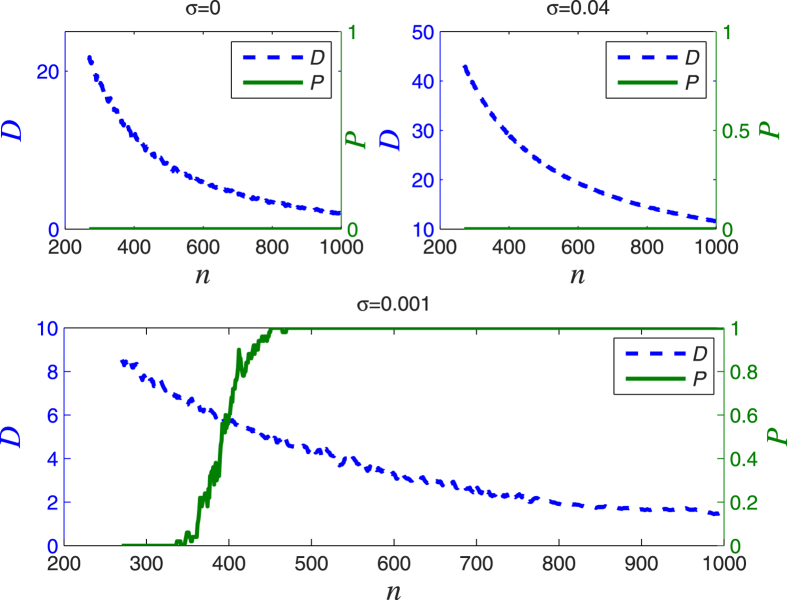
The fitting results for E2 with coupling strength *σ* = 0 corresponding to upper left panel, *σ* = 0.04 corresponding to upper right panel, *σ* = 0.001 corresponding to lower panel, and threshold *T* = 6.5.

In order to investigate the relationship between the number of neurons used for solving Eq. (9), and the successful rate of fitting in the sense of the deviation *D* in Eq. (10), being smaller than a threshold *T*, we show the simulation results for *σ* = 0, *σ* = 0.04, *σ* = 0.001 in Fig. 10, respectively. This figure shows the relationship between the number of neurons used for solving Eq. (9) and producing the modelled signal *S*_*N*_(*t*), and the successful rate with respect to the predefined deviation, where the successful rate is obtained from 200 runs using selected number of neurons. The criterion of successful modelling is the deviation being smaller than *T* = 6.5 (microvolt). We also define the quantity *P* as the percentage of fittings that produce *D* < *T* from all fittings, considering randomly selected neurons in each run. We conclude from Fig. 10 (lower panel) that the successful fitting rate *P* is 100 percent when the number of neurons used is more than 450 and the coupling strength is 0.001, where the CAS phenomenon is present. However, for the coupling strength equal to 0 (Fig. 10, upper left panel) and 0.04 (upper right panel), the fitting fails (*P* = 0, regardless of *n* and *σ*). From these observations, we provide further evidence that the brain operates on a CAS state. That being true, it would imply that the brain, in spite of using very small amounts of energy to maintain the weak connections, is capable of reproducing a remarkable large number of patterns.

### The EEG signal approximation using the neuron network with both electrical and chemical couplings

Previous works show that both electrical and chemical couplings are present in the brain^12^,^13^. The EEG signal modelling using the neuron network with both couplings is analysed and compared with the neuron network with only electrical coupling. The comparison results are shown in Table 1. The results are the statistical value of 30 simulations. The electrical coupling strength *σ* = 0.001, the chemical coupling strength *g* = 0.001, the number of neurons used is *n* = 400, the Laplacian matrix representing the network of electrically coupled neurons (scale-free HR network) **A** is different from the adjacency matrix representing the network of chemically coupled neurons **B**; the excitatory chemical synapses are set by making *V*_*syn*_ = 2. From Table 1, we learn that, under the same conditions, the neuron network with both couplings can model the EEG signal better than the neuron network with only linear coupling. This provides further evidence that CAS is very important to reproduce local brain behaviour (e.g., EEG signals), and that neuron networks with neurons coupled by both synapses provide better modelling, reinforcing the idea that in the brain one needs to have these two types of connections.

## Discussion

In this work, we show that the Hindmarsh-Rose neuron networks, with both chemical and electrical couplings and operating^25^ in a regime that exhibits the so called Collective Almost Synchronisation (CAS), produces an output signal that model very well the EEG signal recorded from four randomly selected regions of the brain. The model is therefore neither subject dependent nor region dependent. This suggests that local regions of the brain could be functioning in a CAS state. This implies that small-scale clusters of neurons in the brain are weakly connected, and that there is a large number of neurons that are almost synchronous. Each local cluster of neurons is roughly decoupled from other brain locations. Neuron networks connected with both types of synapses (electrical and chemical) reproduce better the EEG signals, reinforcing the idea that both connections are fundamental for the working of the brain. This work therefore provides evidence that CAS is present in the brain. Neurons that are in the CAS regime in the local clusters have the same capabilities as our simulated neurons, presenting an infinite number of patterns that appear for small changes in the synaptic couplings. These patterns, in turn, could explain the brain large memory capacity. Our work thus also support the idea that local clusters of neurons are sufficiently decorrelated, so they process information independently. Therefore, we can interpret the EEG signal as a manifestation of a local cluster of weakly connected neurons integrating information and capable of emulating an infinite number of almost periodic oscillatory patterns. A spectral analysis reveals that the power spectrum curves of the EEG signals are reasonably different. However, the same simulated network can model the EEG from every region. Moreover, our simulations show that simulated networks with different topologies and different number of neurons can fit with similar accuracy the experimental EEG signals. Therefore, the success of the model does not rely on any spectral similarities between the simulated network and the EEG signal, but rather on the nodes behaving in the CAS regime.

## Additional Information

**How to cite this article:** Ren, H.-P. *et al*. Weak connections form an infinite number of patterns in the brain. *Sci. Rep.* **7**, 46472; doi: 10.1038/srep46472 (2017).

**Publisher's note:** Springer Nature remains neutral with regard to jurisdictional claims in published maps and institutional affiliations.

## Figures and Tables

**Figure 1:**
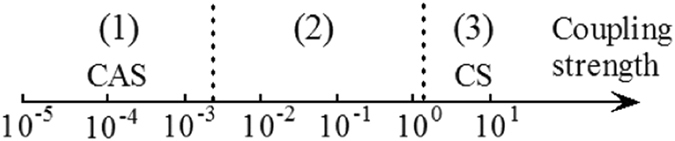
A pictorial representation of the relationship between the strength of neuron network couplings and the behaviour of synchronisation. CAS means Collective Almost Synchronisation; CS means Complete Synchronisation.

**Table 1:** The result of deviation for neuron networks with different coupling types.

			EEG Locations			
			E1	E2	E3	E4
Coupling Types	Electrical coupling	maximum Deviation	1.0429	1.1708	1.0573	1.9172
		mean Deviation	0.9802	1.1315	0.9273	1.5429
		minimum Deviation	0.9203	1.0574	0.8729	1.4957
	Electrical & chemical couplings	maximum	0.3902	0.5133	0.3278	0.6276
		mean Deviation	0.3848	0.5044	0.2937	0.6133
		minimum Deviation	0.3762	0.4912	0.2791	0.5990
